# T-Cell Acute Lymphoblastic Leukemia—Current Concepts in Molecular Biology and Management

**DOI:** 10.3390/biomedicines9111621

**Published:** 2021-11-04

**Authors:** Parveen Shiraz, Waqas Jehangir, Vaibhav Agrawal

**Affiliations:** 1 Blood and Marrow Transplantation/Cell Therapy, Stanford University, Stanford, CA 94305, USA; 2 Avera Medical Group Hematology, Transplant & Cellular Therapy, Sioux Falls, SD 57105, USA; waqas.jehangir@avera.org; 3 Department of Hematology & Hematopoietic Cell Transplantation, City of Hope National Medical Center, Duarte, CA 91010, USA; vagrawal@coh.org

**Keywords:** acute lymphoblastic leukemia, T-cell leukemia, NOTCH, Nelarabine

## Abstract

T-cell acute lymphoblastic leukemia (T-ALL) is an uncommon, yet aggressive leukemia that accounts for approximately one-fourth of acute lymphoblastic leukemia (ALL) cases. *CDKN2A*/*CDKN2B* and *NOTCH1* are the most common mutated genes in T-ALL. Children and young adults are treated with pediatric intensive regimens and have superior outcomes compared to older adults. In children and young adults, Nelarabine added to frontline chemotherapy improves outcomes and end of consolidation measurable residual disease has emerged as the most valuable prognostic marker. While outcomes for de-novo disease are steadily improving, patients with relapsed and refractory T-ALL fare poorly. Newer targeted therapies are being studied in large clinical trials and have the potential to further improve outcomes. The role of allogeneic stem cell transplant (HSCT) is evolving due to the increased use of pediatric-inspired regimens and MRD monitoring. In this review we will discuss the biology, treatment, and outcomes in pediatric and adult T-ALL.

## 1. Introduction

Children, adolescents, and young adults comprise 70% of ALL cases [1]. The incidence of ALL in United States (US) is 1.8 per 100,000 for all age groups and 5 per 100,000 for ages 0–19 [1]. While the incidence in Europe is comparable to the US, data suggest higher incidence in Mexico and other Latin American countries [2,3,4]. Survival in ALL is strongly influenced by age with five-year overall survival being 80% in <50 years and <35% in >50 years [1]. T-ALL comprises 15–25% of ALL cases in children and adults [5,6,7,8,9]. Therefore, T-ALL is primarily a disease of children and young adults and rare in older adults. Sequential accumulation of genomic lesions in the immature T cell progenitors culminates in leukemic transformation and a high proliferative index translates clinically to leukocytosis and extramedullary disease, including large mediastinal/thymic masses and central nervous system (CNS) involvement. The genomic and molecular aberrations seen in T-ALL are distinct from that of B-ALL, yet, up until recently, similar treatment regimens were used for both diseases. The distinction between T-ALL and T-lymphoblastic lymphoma (T-LBL) depends on the degree of bone marrow involvement, with T-ALL cases defined by 20% or more blasts in the bone marrow, whereas T-LBL cases have less than 20% bone marrow blasts with predominance of extramedullary disease [10]. This review will be limited to T-ALL. Early T precursor cell ALL (ETP-ALL) is a distinct subtype arising from immature T-cells and will be discussed in detail. As with B-ALL, outcomes in all age groups, especially for children and young adults, have improved over the past several decades [11,12].

## 2. Genomics and Molecular Biology

We will begin this section with a discussion of normal thymocyte development, including the role of *NOTCH* and *MYC*, followed by a description of the genomic landscape of T-ALL.

### 2.1. Thymocyte Development

Thymocyte development in mice has been extensively studied using in vivo and in vitro models [13]. Uncommitted lymphoid cells from the bone marrow proliferate when stimulated by Interleukin-7 (IL-7) and stem cell factor (SCF) upon entering the thymus [14,15]. These cells express the NOTCH1 receptor and are activated by NOTCH ligands termed “delta-like” and “Jagged”, which are expressed by the thymic epithelial cells [14,16]. NOTCH activation is required to transform lymphoid precursor cells to T-cells and in the absence of NOTCH, these precursor lymphoid cells by default become B cells [17]. It is important to note that these lymphoid precursor cells retain NK and myeloid markers. Early thymocytes that lack surface CD4 and CD8, called double negative (DN) cells, progress through four stages of differentiation labeled DN1, DN2, DN3, and DN4. DN3 cells exhibit pre-TCR (T-cell receptor) composed of pre-Tα and a rearranged TCRβ chain. DN3 cells have high levels of NOTCH signaling [18], which induces marked cell proliferation as they become DN4 cells. Only those DN cells that have successfully rearranged TCRβ chain transform to double positive (DP) cells expressing CD4 and CD8, at which point they cease to proliferate and undergo rearrangement of the TCRα chain to form a complete TCR that can recognize MHC (major histocompatibility complex). Only those DP cells that are capable of recognizing MHC survive (positive selection) and become single positive CD4 or CD8 cells. These single positive CD4 and CD8 cells then face MHC with self-antigens and only those that do not exhibit a strong response to self-antigens survive (negative selection). Positive and negative selection eliminate the majority of thymocytes, leaving behind mature T cells capable of recognizing foreign antigens and tolerant of self-antigens [14].

### 2.2. NOTCH and MYC

NOTCH1 is a transmembrane receptor protein that serves as a transcription factor [14,16]. Once activated by delta-like and Jagged ligands, the intracellular portion is cleaved by gamma secretase, translocates to the nucleus, and activates expression of target genes [14,16,19,20]. FBXW7 directs the intracellular portion of NOTCH1 for degradation, thus terminating NOTCH signaling [14,19,21]. Activating mutations of the *NOTCH* gene or loss of function mutations of the *FBXW7* gene lead to constitutive NOTCH signaling in T-ALL [22,23]. Therapeutic inhibition of this pathway has been studied with NOTCH antibodies and gamma secretase inhibitors, albeit with little success [24,25].

The *MYC* oncogene encodes for transcription factors that regulate genes involved in cell cycle progression, ribosome synthesis, protein translation, and metabolism [26]. *MYC* plays an important role in the self-renewal and differentiation of hematopoietic stem cells [27] as well as in the development of B and T lymphocytes [28,29]. Deregulated *MYC* signaling has been implicated in several tumor types including B and T cell malignancies [30,31]. *MYC* expression is increased in the developing thymic precursor T cells and loss of *MYC* is associated with severely decreased numbers of thymocytes [14,32]. Several preclinical studies of T-ALL have demonstrated high levels of *MYC* expression, which is required for the growth and proliferation of leukemic T-cells [14,33,34,35]. MYC is shown to be downstream of NOTCH1 in the signaling cascade [33]. *NOTCH1* mutations are associated with increased level of *MYC RNA* and conversely *NOTCH1* inhibition decreases *MYC RNA* levels [14]. Additionally, retroviral expression of *MYC* has been shown to rescue leukemic cells from growth arrest induced by NOTCH1 or gamma secretase inhibitors [36]. NOTCH1 activates *MYC* expression via the DNA enhancer sequence NMe (*NOTCH MYC* enhancer) [34]. Enhancer sequences when bound by transcription factors increase the transcription of an associated gene. NMe is occupied by NOTCH1 and directly interacts with the *MYC* promoter and induces *MYC* expression in developing thymocytes and T-ALL cells [34]. NMe knockout mice demonstrate severely decreased thymic cellularity with reductions in immature and mature T cells [34]. In addition, isogenic immune deficient mice fail to develop T-ALL when transplanted with NMe knockout hematopoietic progenitors with retrovirally driven constitutive activation of *NOTCH1* [34]. In addition, NMe deletion after leukemia induction resulted in antileukemic effects and improved survival [34]. Proteosomal degradation of MYC protein which has a short half-life, is mediated by FBXW7 [35]. Therefore, T-ALL cases with *FBXW7* mutations have increased levels of MYC protein [14,35].

### 2.3. Genomic Landscape of T-ALL

T-ALL arises from maturational arrest during thymocyte development with subsequent proliferation, survival, altered metabolism, and enhanced homing. These events are triggered by sequential accumulation of multiple genetic aberrations. Each case of T-ALL has on an average, more than ten genetic aberrations, which disrupt distinct intracellular pathways [23,37], including activation of oncogenic transcription factors, loss of tumor suppressor genes, increased kinase signaling, epigenetic lesions, and defective ribosomal proteins and RNA translation [23,37]. Table 1 enlists pathways and genes involved with the frequency and distribution among children and adults. *CDKN2A*/*CDKN2B* tumor suppressor genes and *NOTCH1* transcription factor gene are the most common altered genes in T-ALL [23,37,38]. Mutations in *NOTCH1* were identified in 50% of the 150 children with T-ALL treated on the ALL-Berlin-Frankfurt-Munster (BFM) 2000 study, and among them, 60% were in the heterodimerization domain, ~20% in the PEST domain, and ~20% in both domains [39]. *NOTCH1* mutational status correlates with the common cortical immunophenotype [39]. Similarly, among 212 adult patients with T-ALL treated on the GRAALL-2003 and -2005 trials, NOTCH1 and FBXW7 mutations were identified in 67% [40]. Genome-wide sequencing techniques have identified more than 100 different genes that are mutated or rearranged in T-ALL [23,41]. Important among them are dysregulated expression of transcriptional factor genes. T-ALL subgroups that correlate with the intra-thymic stage of differentiation have been identified based on the unique and mutually exclusive transcriptional factor involved [23].

*IL7R* and *JAK* mutations are present in 20–30% of T-ALL cases leading to increased JAK/STAT signaling downstream of the IL7 receptor. These mutations are mostly observed in early cortical (*TLX3*/*TLX1*) and ETP-ALL (*HOXA*, *LMO2*/*LYL1*) subgroups [37,42,43,44,45]. Increased IL7R signaling is also noted in cases without these mutations, indicating the presence of other factors activating the IL7R pathway [37,46,47]. Episomal modifications are present in more than 50% of T-ALL cases and include *DNMT3A*, *EED*, *EZH2*, *KDM6A*, *PHF6,* and *SUZ12* [37,48,49]. Non-germline somatic mutations in ribosomal protein genes *RPL5*, *RPL10,* and *RPL22* are seen in 20% of T-ALL cases [37,50]. Oncogenic microRNAs can downregulate tumor suppressor genes such as *IKZF1*, *PTEN,* and *FBXW7* [37]. Relapsed T-ALL exhibits *NT5C2* enzyme gene mutations leading to increased nucleotidase activity and resistance to maintenance chemotherapy drugs 6-MP and 6-TG [37]. The cryptic fusion *NUP214-ABL1* resulting in epigenetic amplification of *ABL1* has been described in 5–10% of T-ALL cases [51,52] rendering them sensitive to inhibition by tyrosine kinase inhibitors Imatinib, Nilotinib, and Dasatinib [53]. *NUP214-ABL1*-mediated proliferation is SRC family kinase LCK-dependent and therefore dual ABL1/SRC kinase inhibitors Dasatinib and Bosutinib may be better suited for the treatment of this subtype of T-ALL [54]. Rare cases of *BCR-ABL1* fusion have also been described [55,56].

**Table 1:** Altered pathways, genes, and their frequencies in T-ALL [23,37,41].

Pathway	Genes	Frequency (Children)	Frequency (Adults)
**NOTCH signaling**	*NOTCH1* *FBXW7*	75% ~25%	>50% 11%
**Cell cycle, tumor suppression**	*CDKN2A/CDKN2B* *CDKN1B*, *CCND3*, *RB1*	>70%	~45–55%
**Transcription**	*TAL1*, *TLX1*, *TLX3*, *LMO2*, *LYL1*, *HOXA*, *NKX2-1*, *BCL11B* and others	>90%	>90%
**Intracellular signaling**	*JAK1*, *JAK3*, *STA5B*, *IL7R*, *KRAS*, *NRAS*, *PTEN*, *PI3KCA*, *FLT3*, *ABL1*	>60%	>60%
**Epigenetic modification**	*DNMT3A*, *EZH2*, *TET2*, *IDH1*, *IDH2*, *SUZ12*, *EP300*, *MLL2*, *WHSC1*	>35%	>60%
**Ribosomal function**	*RPL5*, *RPL10*, *RPL22*	>10%	>10%
**RNA translation**	*mTOR*, *CNOT3*	~9%	~9%

PICALM-MLL10 is the most common fusion protein in T-ALL and is seen in 6–7% of pediatric and adult T-ALL [57]. This fusion results in upregulation of HOXA genes [58] as do MLL-AF6, SET-NUP214, and TCRB-HOXA [59,60]. It is interesting to note that PICALM and HOXA aberrations are also seen in AML [61,62].

### 2.4. Prognostic Significance of Genomic Aberrations in T-ALL

*NOTCH1* mutations correlate with good prednisone response, favorable MRD kinetics, and long-term outcomes in pediatric patients treated on ALL-BFM protocols [39]. In the FRALLE2000T study, among 220 children with T-ALL, the favorable prognostic significance of *NOTCH1*/*FBXW7* mutations was restricted to patients without *RAS*/*PTEN* mutations [63]. In the UKALL2003 trial, among 162 pediatric T-ALL patients, outcomes correlated with the number of mutations, with five-year OS 82, 88, and 100% for *NOTCH1*/*FBXW7* wildtype, *NOTCH1* mutated/*FBXW7* wildtype, and *NOTCH1*/*FBXW7* double mutated, respectively [64]. However, in the UKALLXII/E2993 trial, among 88 adult T-ALL patients, there was no significant difference in disease response based on *NOTCH1* and *FBXW7* mutations [65]. In the GMALL 05/93 and 06/99 trials, among 126 adult T-ALL patients, *NOTCH1* and *FBXW7* mutations were associated with favorable outcomes but only with low expression of ERG and BAALC [66]. High expression of ERG and BAALC has been associated with an immature leukemic phenotype and the above result could indicate a more differentiated leukemia that is susceptible to combination chemotherapy. In the GRAALL-2003 and -2005 trials with 212 adult T-ALL patients, *RAS*/*PTEN* mutations conferred poor prognosis and *NOTCH1*/*FBXW7* mutations conferred favorable prognosis but only in the absence of *RAS*/*PTEN* abnormalities [40]. Other studies have also shown high-risk disease and poor prognosis with *PTEN* mutations [67,68,69]. Mutational loss of *PTEN* has been linked to resistance to pharmacological inhibition of NOTCH1 with γ-secretase inhibitors [70].

## 3. Immunophenotypic Classification

The immunophenotypic characteristics of T-ALL lymphoblasts reflects the intra-thymic stage at which differentiation arrest takes place. The WHO 2008/2016 criteria for T-lineage assignment uses strong cytoplasmic (Cy) or surface (S) CD3 [10]. The EGIL classification similarly defines T-ALL by Cy or S expression of CD3 and subgroups T-ALL as follows [71]:

1. Pro-T: CD7+, CD2−, CD5−
2. Pre-T: CD2+, CD5+/−, CD8+/−
3. Cortical-T: CD1a+
4. Mature-T: sCD3+, CD1a−

**Figure 1:**
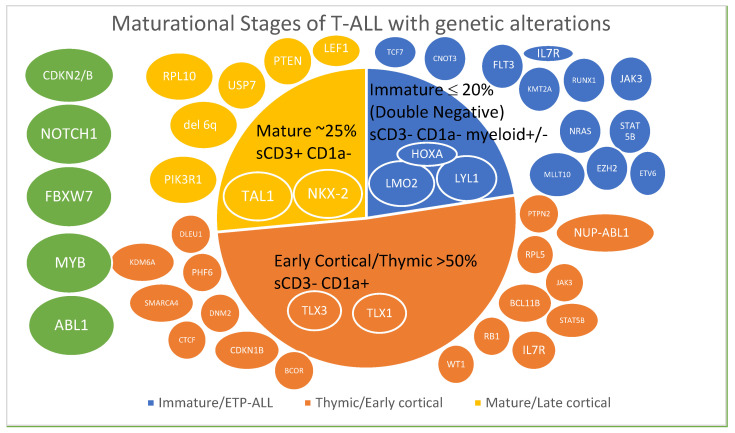
Maturational stages of T-ALL color-coded with associated genetic alterations [23,37]. Transcription factors affected are indicated in ovals within the subtype and gene alterations are depicted outside the subtypes. Genes in green ovals are altered across different subtypes.

Another classification was based on immunophenotype of patients treated in three consecutive GMALL studies, 05/93, 06/99, and 07/2003. T-ALL was defined by the presence of cyCD3 and CD7 and was subclassified as follows [8]. Figure 1 depicts the distribution of cases in these three studies.

1. Early T-ALL—sCD3−, CD1a−
2. Thymic T-ALL—sCD3−/+, CD1a+
3. Mature T-ALL—sCD3+, CD1a−

Although the latter classification is more commonly used for immunophenotypic subtyping, the prognostic impact of either system is limited, with the exception of Early T-ALL, the prognostic significance of which is described below.

### Early T Precursor Cell ALL (ETP-ALL)

ETP-ALL is a subtype of T-ALL with distinct genetic and immunophenotypic characteristics. It comprises 12–20% of T-ALL cases in children and adults [72,73]. The immunophenotype of ETP-ALL is defined as CD1a−, CD8−, CD5−/weak along with the expression of one or more myeloid or stem cell markers such as CD117, CD34, HLA-DR, CD13, CD33, CD11b, and CD65 [72]. The leukemic cells derive from immature thymocytes that retain stem cell and myeloid lineage characteristics. Defects in transcriptional factors LMO2, LYL1, and HOXA and mutations in *IKZF1* and *MED12* and rearrangement of *NUP98* as well as myeloid malignancy mutations such as *FLT3*, *WT1*, *EZH2*, *RAS*, *RUNX1,* and *NPM1* are also seen [23,46,74,75,76]. In the GRAALL-2003 and -2005 studies, HOXA overexpression was associated with poor prognosis in adults with ETP-ALL. The five-year OS was 31% and 74% in HOXA positive and negative cases, respectively [77]. In this study, among the non-ETP cases, there was no difference in outcomes, with five-year OS being 66% and 57% in HOXA positive and negative patients, respectively. Compared to non-ETP, ETP leukemic cells tend to lack molecular markers for polymerase chain reaction (PCR) detection of minimal/measurable residual disease (MRD), be steroid resistant, and patients are more likely to present with CNS and extramedullary disease and have poor response to induction [78]. However, the use of response-based risk stratification and therapy intensification was shown to abrogate the poor prognosis of adult ETP-ALL in the GRAALL-2003 and -2005 studies [79]. On the other hand, adults with ETP-ALL treated on the HyperCVAD protocol had poor outcomes [73]. In children and young adults with ETP-ALL, although earlier studies reported poor outcomes compared to non-ETP-ALL [72], with the current use of intensified pediatric regimens, outcomes are comparable in both groups [80].

## 4. Risk Stratification and Measurable Residual Disease (MRD)

### 4.1. Children’s Oncology Group (COG) Risk Groups

The COG risk stratifies pediatric T-ALL as depicted below [81]. M1, 2, and 3 marrow indicate <5%, 5–25%, and >25% blasts, respectively. CNS1 indicates <5 WBC/μL with no blasts in CSF. MRD was determined by flow cytometry at a central lab.

**Standard risk**—Day 29 M1 marrow and MRD < 0.01%, CNS1, no testicular disease and no steroid pretreatment

**Intermediate risk**—Day 29 M1 or M2 marrow and MRD >/= 0.01%, end of consolidation MRD < 0.1%, any CNS/testicular disease status, any steroid pretreatment status

**Very high risk**—Day 29 M3 marrow or end of consolidation MRD >/= 0.1%

### 4.2. UKALL Risk Stratification

The UKALL 2003 study risk stratified ALL patients ages 1–24 years using three parameters [82]:

1. National Cancer Institute (NCI) risk criteria—NCI standard risk—age < 10 years and WBC < 50,000/μL, NCI high risk—age ≥ 10 years and WBC ≥ 50,000/μL
2. Cytogenetics—MLL gene rearrangement, hypodiploidy and iAMP21 were considered high risk
3. Response to induction therapy in age < 16 years—day 8 or 15 bone marrow with < or >25% blasts

Based on above parameters, three clinical risk groups were identified:

1. Clinical standard risk—NCI standard risk with <25% blasts at day 15 after induction and without high-risk cytogenetics
2. Clinical intermediate risk—NCI high risk with <25% blasts at day 8, all patients ≥ 16 years irrespective of day 8 or 15 marrow response
3. Clinical high risk—high-risk cytogenetics as noted above, NCI high risk with >25% blasts at day 8, NCI standard risk with >25% blasts at day 15

Clinical standard and intermediate risk patients were further stratified based on MRD response (determined at 10^−4^ range as assessed by PCR of Ig or TCR gene rearrangement), at the end of induction and consolidation.

1. MRD low risk—MRD undetectable before start of interim maintenance, with undetectable or detectable at <0.01% MRD at the end of induction
2. MRD intermediate risk—MRD could not be measured or MRD positive at <0.01% before start of interim maintenance
3. MRD high risk—MRD at least 0.01% at the end of induction

### 4.3. MRD

Although clinical risk factors such as age, WBC at presentation, presence of CNS disease [11], and genetic abnormalities such as *RAS* and *PTEN* mutations [40] have prognostic significance, MRD remains the single most important prognostic indicator in pediatric and young adult T-ALL as demonstrated in multicenter cooperative group trials [83,84]. MRD can be measured by multi-color flow cytometry or polymerase chain reaction (PCR) of clonal T-cell receptor (TCR) gene rearrangements with a sensitivity of 10^−4^ for both methodologies [85]. Unlike B-ALL where a high percentage of pediatric and young adult patients achieve early MRD negativity, more than 80% of T-ALL patients remain MRD positive at the end of induction as demonstrated in the AIEOP-BFM-ALL 2000 study [86]. In this study, the seven-year EFS was 91, 80, and 50% with negative MRD after induction, negative MRD after consolidation, and persistent MRD after consolidation, respectively [86]. In the UKALL 2003 study, there was no significance reduction in the five-year EFS in low MRD-risk patients who received one versus two delayed intensification regimens, suggesting that treatment reduction is feasible in this risk subgroup [82]. While end of induction MRD may help identify patients eligible for reduced intensity therapy, end of consolidation (EOC) MRD can identify patients at high risk of relapse who would benefit from allogeneic stem cell transplant (HSCT) [86,87,88,89].

## 5. Frontline Treatment

While multiagent chemotherapy regimens remain the cornerstone of first line therapy in children and adults, the regimens used, and the outcomes observed in T-ALL differ between the two age groups and will therefore be discussed separately. Until the advent of Nelarabine, B-ALL and T-ALL patients were treated similarly in clinical trials. For pediatric ALL as a whole, five-year overall survival (OS) has improved from <20% in the 1960s to >90% since 2000, and for T-ALL the 5-year OS reached ~80% in 2000 [11].

### 5.1. Children, Adolescents, and Young Adults (AYAs)

**Figure 2:**
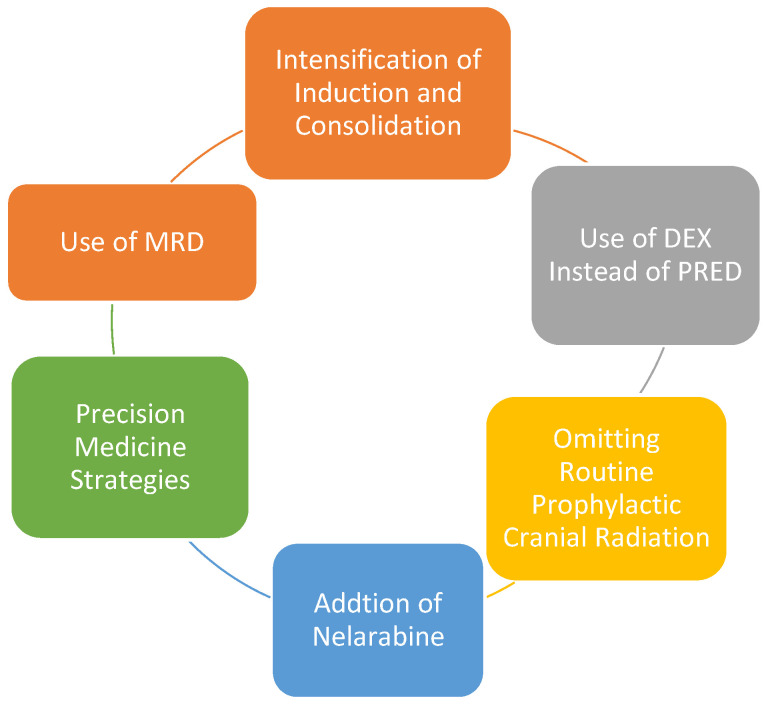
Strategic Advances in the Management of Pediatric T-ALL.

The past several decades has seen a steady improvement in the survival of children and AYAs with T-ALL primarily due to incremental and strategic changes in multiagent chemotherapy regimens in children and the adoption of pediatric-inspired protocols in young adults [11,90]. We will discuss treatment in this age group in the context of the following advances which are depicted in Figure 2. Table 2 summarizes the recent studies.

1. **Intensification of induction and consolidation**—This includes using a four-drug induction regimen containing a steroid (dexamethasone or prednisone), anthracycline, vincristine, and asparaginase and an augmented Berlin-Frankfurt-Munster (BFM)-based consolidation regimen using cyclophosphamide [9,87,91,92,93].
2. **Use of dexamethasone (DEX) instead of prednisone (PRED) during induction**—Both DEX and PRED as the steroid of choice during induction in pediatric ALL have been studied in several trials. DEX being more potent and capable of CNS penetration, has been shown to decrease overall and CNS relapse. In the AIEOP-BFM ALL 2000 study, a significant survival benefit was observed with DEX for T-ALL patients [94]. Similarly, in the UK MRC ALL97 and ALL97/99, DEX demonstrated a lower risk of isolated CNS relapse and improved EFS compared to prednisolone [95,96]. Subsequently, the UKALL 2003 used only DEX and the T-ALL patients had superior outcomes with three-year EFS 86% and OS 90% [97]. The use of DEX was shown to have a higher risk of infections and other toxicity [94] and warrants careful monitoring of these patients. Although the COG AALL0434 reported excellent outcomes using PRED in T-ALL, in this study, all intermediate risk (IR) and high risk (HR) patients received cranial radiation, which confounds the lack of CNS relapse with PRED.
3. **Omitting routine prophylactic cranial radiation (CRT)**—In order to decrease toxicity and yet maintain efficacy, several cooperative group studies in the US and Europe omitted prophylactic CRT and intensified systemic and intrathecal therapy in pediatric ALL and have shown this strategy to be effective with low rates of CNS relapse between ~3–8% in T-ALL patients and comparable to those who received CRT [98,99,100,101,102,103]. The intensification strategies used varied between the different trials and included combinations of triple intrathecal therapy, high dose methotrexate, higher asparaginase doses, and use of DEX, but nevertheless, demonstrated that prophylactic CRT can be safely eliminated in most pediatric patients with T-ALL.
4. In the context of using **DEX and minimizing CRT,** the COG AALL1231 combined both these strategies [104]. This was a phase three trial that randomized T-ALL/LL patients ages 1–30 years, to a modified augmented BFM backbone with or without Bortezomib during induction and delayed intensification (DI). Only the very high risk (VHR) patients as defined by day 29 M3 marrow, EOC MRD > 0.1% or overt CNS leukemia (CNS3), received cranial radiation. Following the MRC strategy, DEX was used instead of PRED and an extra dose of PEG-ASP was added to induction and DI. While standard risk (SR) and intermediate risk (IR) patients had improved three-year EFS with Bortezomib (>90% vs. 85% for both groups, *p* < 0.05), VHR patients did worse with Bortezomib with three-year EFS of 37% vs. 6% (*p* = 0.03) [104]. However, the study closed early (when Nelarabine was shown to improve DFS in AALL0434), and it was not sufficiently powered to determine the effect of adding Bortezomib to chemotherapy backbone to the entire T-ALL cohort irrespective of risk status.
5. **Nelarabine** is a purine nucleoside analog, a prodrug of Ara-G, and cytotoxic to T lymphoblasts. It has been shown to have single agent activity in relapsed T-ALL with high CNS penetration and therefore has potential to decrease CNS relapse, but this comes with the risk of CNS toxicity [105]. Therefore, Nelarabine should not be used with IT chemotherapy and cannot be used in patients with active CNS disease, as these patients will be receiving IT chemotherapy.
6. **Addition of Nelarabine and comparison of high dose (HD) versus Capizzi (escalating doses) methotrexate (C-MTX)** were tested in a randomized fashion in the COG AALL0434 study. Prior to COG AALL0434, the COGAALL0232 had demonstrated that in B-ALL, HD-MTX and DEX improved outcomes compared to C-MTX and PRED [106]. Since disease sensitivity to MTX can vary between B and T-ALL, the COG tested these two strategies in T-ALL in the AALL0434 trial [80]. In addition, since Nelarabine was shown to have superior activity in the relapsed/refractory setting, use of Nelarabine in the upfront setting was tested in a randomized fashion. As a result, AALL0434 had a 2 × 2 factorial design comparing HD-MTX to C-MTX with and without Nelarabine added to the BFM backbone. From 2007 to 2014, this study enrolled 1562 patients with T-ALL, ages 1–31 years, and used an augmented BFM regimen with PRED as steroid and with a 2 × 2 randomization to receive either an escalating dose of MTX (C-MTX) or HD-MTX. All patients with IR and HR disease also received prophylactic CRT and were randomized to receive or not receive Nelarabine. In contrast to the B-ALL results from the COG AALL 0232, where HD-MTX had improved outcomes, C-MTX produced better outcomes in T-ALL with five-year DFS of 91% vs. 83%, *p* = 0.04 and OS 93% vs. 89%, *p* = 0.04 [107]. While the addition of Nelarabine improved five-year DFS (88% vs. 82%, *p* = 0.03), the improvement in five-year OS (90% vs. 88%, *p* = 0.168) did not reach statistical significance [80]. Overall, the five-year DFS was highest with C-MTX with Nelarabine and lowest with HD-MTX without Nelarabine (91% vs. 78%, *p* = 0.01). Another significant finding from this trial was the decrease in CNS relapse rate with Nelarabine (1.3% vs. 6.9%, *p* = 0.0001). In the context of Nelarabine, the UKALL 14, is an ongoing randomized phase III trial, where adult T-ALL patients are randomized to receive standard chemotherapy with or without Nelarabine (NCT01085617). In addition, the GRAALL-2014/T is a multicenter study of risk-adapted treatment for T-ALL in young adults ages 18–59 years, evaluating the efficacy of a Nelarabine-based consolidation and maintenance in high-risk patients (NCT02619630).
7. **Role of allogeneic hematopoietic stem cell transplant (HSCT) in children with T-ALL**—with the high rates of long-term progression free and overall survival achieved with contemporary, intensive pediatric regimens described above, the indications for HSCT in children with T-ALL is decreasing. Persistence of MRD at 10^−3^ or 10^−4^ post consolidation remains the primary indication for HSCT. While HSCT remains an option in very high-risk (VHR) disease (Day 29 M3 marrow, CNS3) [103], many experts recommend using EOC MRD as the primary indication for HSCT [108]. ETP-ALL alone is not considered an indication for HSCT in children as long as they can achieve MRD negativity post consolidation [108].
8. **Incorporating precision medicine strategies**—ALLTogether1 is a prospective observational study designed by seven cooperative groups in Europe (NOPHO, UKALL, DCOG, COALL, BSPHO, SHOP, and SFCE) using a common treatment protocol based on a novel personalized algorithm using clinical characteristics, genetic changes, and response to treatment for patients ages 1–45 years with newly diagnosed B and T-ALL (NCT03911128). Total Therapy XVII is the equivalent study in the US enrolling B and T-ALL patients with the objective of using novel precision medicine strategies based on genomic features of the leukemia and tailoring treatment for individual risk groups. The T-ALL cohort would receive, in addition to combination chemotherapy, a targeted agent such as Dasatinib, Bortezomib, or Ruxolitinib depending on the respective aberration detected (NCT03117751).

**Table 2:** Recent studies in pediatric and AYA T-ALL.

No.	Name of Trial Years of Enrollment	No. of T-ALL Patients Age Range (y)	Steroid Used	Cranial Radiation	Novel Agent Used	DFS and OS in T-ALL Patients	Ref
1.	CALGB 10403 2007–2012	71 17–39 y	Pred *	Yes—in all T-ALL	None	3 y DFS 66% 3 y OS 73%	[7]
2.	COG AALL0434 2007–2014	1562 1–31 y	Pred	Yes—in IR and HR groups **	Nelarabine	5 y EFS 83% 5 y OS 89%	[80]
3.	COG AALL1231 2014–2017	847 1–30 y	Dex	Only in VHR ^†^ group	Bortezomib	3 y EFS 83% 3 y OS 86%	[104]
4.	NOPHO ALL2008 2008–2014	231 1–45 y	Dex	No	None	5 y EFS 74% 5 y OS 75%	[109]
5.	Total therapy XVI 2007–2017	104 0–18 y	Pred	No	None	5 y EFS 81% 5 y OS 87%	[103]

* Pred–prednisone, Dex–dexamethasone ** IR—intermediate risk, HR—high risk ^†^ VHR—very high risk: Day 29 M3 marrow, EOC MRD > 0.1%, CNS3.

### 5.2. Use of Pediatric-Inspired Regimens in Young Adults

Inspired by the superior outcomes achieved in childhood ALL with intensive multiagent chemotherapy regimens, adult oncology groups adopted and prospectively studied these regimens in young adult ALL and demonstrated both feasibility and efficacy [110,111,112,113]. The Cancer and Leukemia Group B (CALGB) 8811 used a five-drug induction regimen similar to CCG-192P and a BFM-based consolidation [111] with higher doses of cyclophosphamide and earlier and more extensive use of L-asparaginase. Patients with T-ALL comprised 22% of the cohort and achieved a comparable three-year DFS (57%) to B-ALL patients. Other groups including GRAALL and PETHEMA have shown similar outcomes using pediatric regimens in young adult patients [114,115,116,117]. Encouraged by these positive results demonstrating feasibility and efficacy, the CALGB 10403 sought to further demonstrate that pediatric protocols can be safely administered to young adults by oncologists treating adult patients [7]. From 2007 to 2012, CALGB 10403 enrolled 318 adolescent and young adults (AYAs) ages 17–39 years, with B- and T-ALL, with the latter constituting 24% of the cohort and used a regimen that was identical to the C-MTX arm of COG AALL0232 with four intensive courses that included induction, consolidation, interim maintenance, delayed intensification, and a prolonged maintenance. PRED was used during induction and DEX during delayed intensification and maintenance. Patients with T-ALL received 24 Gy prophylactic cranial irradiation. There were no significant differences in the outcomes between B and T-ALL, with three-year DFS and OS being 66 and 73%, respectively, for the whole cohort [7]. Most experts recommend a pediatric-inspired regimen as the preferred choice in AYAs with Ph-negative B-ALL and T-ALL [118].

### 5.3. Older Adults with T-ALL

Clinical data on the outcomes of older adults with T-ALL are sparse. The largest study of older adults with ALL was conducted by GMALL, in which 268 patients 55–85 years with ALL were enrolled and treated with pre-phase, induction, consolidation, reinduction, and prolonged maintenance, along with triple chemotherapy CNS prophylaxis. T-ALL comprised 15% of the cohort. OS at five years was 23% for the whole cohort and immunophenotype did not correlated with OS [119].

HyperCVAD is commonly used in adults with ALL. In a study from MDACC, 40 patients with T-ALL, ages 18–78 years were treated with hyperCVAD + Nelarabine, and three-year OS was 62%. Outcomes were not analyzed by age. When sub-grouped by immunophenotype, ETP-ALL (*n* = 15), appeared to have worse outcomes with three-year OS of 50%, although this difference was not statistically significant (*p* = 0.59), likely due to low sample number [120]. Two studies published outside MDACC have reported poor outcomes in adult T-ALL with hyperCVAD [121,122].

In the UKALLXII/ECOG2993, 356 out of 1643 ALL patients (22%), ages 15–59 years, were designated as T-ALL based on intracytoplasmic CD3. T-ALL comprised 38 and 10% of ALL in patients ages 20–29 years and >50 years, respectively, demonstrating the rarity of this disease in the older patients [113]. The five-year OS was 48% for T-ALL of all ages and not statistically different from B-ALL. However, in patients older than 50 years, five-year OS was only 27% compared to >50% for <30 years.

In conclusion, older patients with T-ALL fare worse likely due to a combination of adverse risk disease biology and higher complications from treatment related to underlying comorbidities. More studies in this subgroup are required to tailor treatment to fit disease and patient profile.

### 5.4. Role of Allogeneic Stem Cell Transplant in Adults with T-ALL

While persistent MRD is the primary indication for HSCT in children with T-ALL, adults with high-risk T-ALL fare poor and therefore have a lower threshold to transplant. Indications for HSCT in adults include high-risk disease at presentation (WBC > 100 k/μL, CNS or other extramedullary disease, ETP-ALL, complex karyotype), poor response to induction, persistence of MRD, and of course, relapsed disease with CR2 or beyond [123,124]. The UKALLXII/ECOG2993 trial studied 356 T-ALL patients, of which, 110 had a sibling donor. HSCT was associated with lower relapse (25% vs. 51%, *p* < 0.0001), higher non-relapse mortality (22% vs. 12%, *p* = 0.02), and improved five-year OS (61% vs. 46%, *p* = 0.02). Among 57 patients with ETP-ALL treated in the GMALL studies (05/93-07/03), ~60% received HSCT in first CR and derived benefit with OS being comparable to non-ETP patients [75]. Intensity of conditioning regimen in older patients has been studied extensively. The increased toxicity and NRM of myeloablative conditioning needs to be considered against the potentially increased relapse rate with reduced intensity conditioning [125].

## 6. Relapsed/Refractory (R/R) Disease and Targeted Therapies

Both children and adults with relapsed disease have poor outcomes. OS rates in children are ~25% [126,127] and similar in adults as well [128].

In children with relapsed disease, re-induction is recommended followed by HSCT if the patient achieves remission [129]. The UKALL R3 multidrug reinduction regimen produced better outcomes with Mitoxandrone compared to idarubicin with three-year PFS and OS of 65 and 69%, respectively [130]. NECTAR (NCT00981799) was a phase 1 study of Nelarabine with etoposide and cyclophosphamide in patients 1–21 years of age with T-ALL and T-LL in first relapse. Among nine patients with T-ALL, 44% had a response rate.

The BCL2 inhibitor Venetoclax has been studied in combination with the BCL-X and BCL-2 inhibitor Navitoclax in a phase 1 study in children and adults with relapsed/refractory ALL, of which, 19 patients had T-ALL [131]. The overall CR was 60% and 28% proceeded to transplant or CAR-T cell therapy [131]. There are case reports of Venetoclax with Decitabine [132,133] and a case report of Venetoclax with chemotherapy [134] demonstrating efficacy in patients with relapsed ETP-ALL allowing for consolidation with HSCT. The authors themselves have successfully treated a young adult patient with ETP-ALL who relapsed after myeloablative double umbilical cord transplant, achieved an MRD negative remission with Venetoclax and Decitabine, and has proceeded to a second HSCT with reduced intensity conditioning and a haploidentical donor. The combination of Venetoclax and Bortezomib was shown to be effective in a case series of three patients, with two of them achieving cytogenetic remission and proceeding to HSCT at the eight-month follow-up [135].

The COG AALL07P1 was a phase 2 trial in children with relapsed ALL and LL, of which, 22 were T-ALL. Reinduction regimen included chemotherapy with Bortezomib. CR2 rate was 68% in T-ALL [136]. Following this encouraging response, the COG AALL1231 added BOR to induction in a randomized fashion and the results are as described in Section 5.1.

Daratumumab and Isatuximab, monoclonal antibodies directed against CD38, have safety profile in humans based on clinical studies in multiple myeloma [137,138]. Preclinical models and case reports have demonstrated the efficacy of DARA in T-ALL. Based on these data, a phase 2 study is underway to evaluate the safety and efficacy of Daratumumab added to standard chemotherapy in patients ages 1–30 years with relapsed/refractory B and T-ALL and LBL (NCT03384654). Primary endpoint is complete remission after one cycle for T-ALL and two cycles for B-ALL. Isatuximab is being studied with chemotherapy in phase 1/2 study (NCT03817320).

*NUP214-ABL1* fusion resulting in *ABL1* amplification has been described in 5–10% of T-ALL cases [51,52], rendering them sensitive to tyrosine kinase inhibitors Imatinib, Nilotinib, and Dasatinib [53]. The first case report of a young adult with *NUP214-ABL1* positive T-ALL achieving complete remission with single agent Dasatinib was reported in 2009 [139]. Another case report of a pediatric T-ALL with ABL1 amplification achieving complete remission after the addition of Dasatinib to chemotherapy was reported in 2012 [140]. Although rare, a single case was reported of a young adult with ETP-ALL with *NUP214-ABL1* fusion successfully treated with Dasatinib added to chemotherapy [141]. There are also case reports of T-ALL with *BCR-ABL1* fusion [55,56].

Given the preclinical efficacy of targeting cell cycle regulators CDK4 and 6 [142], the COG launched AINV18P1 (NCT03792256). Patients aged 1–30 years with relapsed B or T-ALL or LL receive the CDK4/6 inhibitor Palbociclib initially as a single agent and subsequently with chemotherapy. Preliminary results demonstrate safety and the expansion phase of this trial is ongoing [143]. Last but not the least, chimeric antigen receptor (CAR) T cell therapy has now entered the realm of T-ALL. A phase 1 pilot study of CD7 targeting CAR-T has demonstrated lack of fratricide and CR in five out of eight enrolled patients with minimal toxicity [144].

Preclinical data demonstrate activation of the JAK/STAT signaling pathway in ETP-ALL and the efficacy of the JAK inhibitor Ruxolitinib in xenograft models of ETP-ALL [45]. The PI3K/AKT/mTOR pathway is shown to be activated in T-ALL due to PTEN inactivation, and inhibition of this pathway has demonstrated efficacy in preclinical models of T-ALL [145]. Combined targeting of CDK and mTOR is being studied in a phase 1 trial of Ribociclib with Everolimus and DEX in patients ages 1–30 years with relapsed/refractory B and T-ALL (NCT03740334). While the development of non-specific γ-secretase inhibitors was hampered by toxicity, the presenilin-1 (PSEN1) subunit γ-secretase inhibitor has demonstrated activity in preclinical models of *NOTCH*-mutated T-ALL with minimal toxicity and may have therapeutic potential [25].

## 7. Conclusions

Tremendous progress has been made in uncovering the genetic underpinnings of T-ALL. Yet, several more defects remain to be discovered. Comparable progress in outcomes has been achieved with combination chemotherapy regimens especially in children and young adults, and yet, toxicities of these regimens remain significant. MRD has emerged as the most prominent risk factor to act upon. As we enter the era of targeted therapies, one can hope to improve outcomes further especially in older adults and decrease toxicity and improve long-term quality of life for children and young adults. Attempts to increase enrollment in clinical trials and eliminate racial and geographic disparities in access to high volume centers are essential for improving outcomes and should be emphasized.

## Data Availability

Not applicable.
